# Chikungunya Particle and RNA Induce Mechanical and Heat Hypersensitivities in a TRPV1-Dependent Manner

**DOI:** 10.3390/biom15020171

**Published:** 2025-01-23

**Authors:** Liziane C. M. da Silva, Andressa C. dos Santos Maia, Nágila C. F. de Sousa, Catielen P. Pavi, Beatriz P. Savi, Seigo Nagashima, Samara Damasceno, Ayda H. Schneider, Lucas Z. Mascarin, João F. S. Rodrigues, Cinara R. A. V. Monteiro, Izabella T. Silva, Gislaine Fongaro, Valério Monteiro-Neto, Maria R. Q. Bomfim, Thiago M. Cunha, João de Sousa Valente, João B. Calixto, Lúcia de Noronha, Susan D. Brain, Elizabeth S. Fernandes

**Affiliations:** 1 Programa de Pós-Graduação em Biotecnologia Aplicada à Saúde da Criança e do Adolescente, Faculdades Pequeno Príncipe, Curitiba 80230-020, PR, Brazil; lizianecms@gmail.com (L.C.M.d.S.); andressamaia0607@gmail.com (A.C.d.S.M.); 2 Instituto de Pesquisa Pelé Pequeno Príncipe, Curitiba 80250-060, PR, Brazil; 3 Programa de Pós-Graduação, Universidade CEUMA, São Luís 65075-120, MA, Brazil; nagila.fialho@discente.ufma.br (N.C.F.d.S.); joao.rodrigues@facsur.net.br (J.F.S.R.); mrqbomfim@gmail.com (M.R.Q.B.); 4 Departamento de Microbiologia, Imunologia e Parasitologia, Centro de Ciências Biológicas, Universidade Federal de Santa Catarina, Florianópolis 88040-900, SC, Brazil; catielen.p@gmail.com (C.P.P.); beasavis2@gmail.com (B.P.S.); lucas.mascarin@gmail.com (L.Z.M.); izabella.thais@ufsc.br (I.T.S.); gislainefongaro@gmail.com (G.F.); 5 Escola de Medicina e Ciências da Vida, Pontifícia Universidade Católica do Paraná, Curitiba 80215-901, PR, Brazil; seigo.nagashima@pucpr.br (S.N.); lnno.noronha@gmail.com (L.d.N.); 6 Faculdade de Medicina de Ribeirão Preto, Universidade de São Paulo, Ribeirão Preto 14049-900, SP, Brazil; samarad.bio@gmail.com (S.D.); ayda.hs@usp.br (A.H.S.); thicunha@fmrp.usp.br (T.M.C.); 7 Programa de Pós-Graduação em Ciências da Saúde, Universidade Federal do Maranhão, São Luís 65085-040, MA, Brazil; cinara.monteiro@discente.ufma.br (C.R.A.V.M.); valerio.monteiro@ufma.br (V.M.-N.); 8 The School of Cardiovascular and Metabolic Medicine (South Bank), King’s College London, London SE1 9NH, UK; joaosousavalente@gmail.com (J.d.S.V.); sue.brain@kcl.ac.uk (S.D.B.); 9 Centro de Inovação e Ensaios Pré-Clínicos, Florianópolis 88056-000, SC, Brazil; joao.calixto@cienp.org.br

**Keywords:** Chikungunya, viral RNA, arthralgia, TRPV1, nociception

## Abstract

Chikungunya virus (CHIKV), the causative agent of the chikungunya fever, is an alphavirus widely transmitted by the bite of the female mosquito of the genus *Aedes* sp., especially in tropical and subtropical regions. Brazil is the country most affected by the microorganism. CHIKV classically induces articular pain, which can become long lasting for even years in a great number of the infected individuals, reducing their quality of life. The mechanisms of CHIKV-induced pain are poorly understood, but recent evidence indicated a role for the transient receptor potential vanilloid 1 (TRPV1) in this pathology. Herein, we assessed the ability of intra-articularly injected inactivated CHIKV or its RNA to trigger nociception in mice. Both stimuli induced bilateral secondary hyperalgesia to mechanical and heat stimuli. These responses were attenuated by TRPV1 ablation or antagonism. Joint structural alterations and increased cartilage TRPV1 protein expression were detected in the ipsilateral knee joints injected with either CHIKV or viral RNA. However, the lack of this receptor did not influence the histological changes triggered by CHIKV or RNA. The results further support the role of TRPV1 in CHIKV-induced pain and highlight its importance in the chronic phase of the disease.

## 1. Introduction

The Chikungunya virus (CHIKV) disease represents a significant burden to countries of tropical and subtropical regions of the globe, especially in South America. The latest report by the European Centre for Disease Prevention and Control (ECDC) estimates that nearly 480,000 individuals were infected by the virus from January to November 2024 [1]. The same report stated that the most affected countries were Brazil, Paraguay, Argentina and Bolivia, and it also indicated an elevated risk for outbreaks in continental Europe due to favorable environmental conditions. CHIKV is a single-stranded RNA alphavirus [2,3] transmitted through the bite of female *Aedes* sp. mosquitoes commonly associated with painful and long-lasting arthralgia [4,5,6]. Different mechanisms have been suggested for CHIKV-induced articular disease including the virus’s ability to infect and replicate in macrophages, synovial and skeletal muscle cells and to persist in joint tissues [7,8], thus contributing to disease chronification. Also, inflammatory mediators such as cytokines were suggested to influence pain severity and persistence [9,10].

Most recently, the transient receptor potential vanilloid 1 (TRPV1)—a well-known pain transducer [11,12] and temperature sensor [13], was implicated in the acute increased nociception elicited by CHIKV or its envelope protein E2 [14]. The study demonstrated that neuronal TRPV1 contributes to this response following sensitization by a yet unknown component. Moreover, the functional expression of TRPV1 on macrophages is involved in the replication of CHIKV in these cells and cytokine production following infection [15]. Of note, TRPV1 is also functionally expressed on synoviocytes, chondrocytes, osteoclasts and osteoblasts [16,17,18] and its importance as a molecular target in different chronic painful disorders including arthritic conditions has been well documented [19,20].

Herein, we used TRPV1 knockout mice and antagonism to assess its contribution to the chronic nociceptive responses and joint damage triggered by the intra-articular (i.art.) injection of inactivated CHIKV or its RNA. TRPV1 was found to mediate the development and maintenance of viral-induced bilateral hypersensitivities to mechanical and heat stimuli.

## 2. Materials and Methods

### 2.1. CHIKV Sample

CHIKV was obtained from a serum sample of an infected patient from Maranhão (Brazil) in 2016, isolated and identified (GenBank deposit no MT075546.1) following approval by the Universidade CEUMA’s Ethics Committee (CEP-UNICEUMA; protocol no 2.498.658; approved on 18 February 2018); the study was conducted in accordance with the Declaration of Helsinki. The virus underwent 7 passages in Vero E6 cells cultured in DMEM plus 10% fetal bovine serum (Thermo Fisher Scientific, Inc., Waltham, MA, USA). CHIKV (10^10^ plaque-forming units (PFUs) per milliliter (mL)) was inactivated by exposure to UV light for 120 min, centrifuged and resuspended in 1 ml sterile phosphate-buffered saline (PBS; Sigma-Aldrich, São Paulo, SP, Brazil). Virus inactivation was confirmed in Vero E6 cell culture for 10 days. Viral RNA was extracted (QIAmp Viral RNA MiniKit, Qiagen, São Paulo, SP, Brazil) and quantified in nanograms (NanoDrop; Thermo Fisher Scientific). Inactivated CHIKV and viral RNA were aliquoted and kept at −80 °C for further use.

### 2.2. Mice

Male and female C57BL/6 mice (total n = 35 mice, 21 males and 14 females; 10–12 weeks old) obtained from the Instituto Carlos Chagas (Fundação Osvaldo Cruz, FIOCRUZ, Curitiba, PR, Brazil) were used. Wild-type (WT; total n = 18, 9 males and 9 females) and transient receptor potential vanilloid 1 (TRPV1; total n = 18, 9 males and 9 females) knockout (KO) animals (10–12 weeks old) were obtained from the Universidade de São Paulo (USP, Ribeirão Preto, SP, Brazil). Experimental protocols were approved by the Animal Use Ethics Committee of the Instituto de Pesquisa Pelé Pequeno Príncipe (IPPPP; protocol number 049/2020; approved on 1 September 2020) and USP (protocol number 1116/2022; approved on 26 September 2022) and were in accordance with the Brazilian Council of Animal Experimentation Control (CONCEA) guidelines and the Animal Research: Reporting in vivo Experiments (ARRIVE) guidelines [21]. Intra-articular (i.art.) injections were performed in mice previously anaesthetized (ketamine (50 mg/kg; Syntec, Barueri, SP, Brazil) + xylazine (1 mg/kg; Syntec) for C57BL/6 or isoflurane (2% (Cristália, Itapira, SP, Brazil)/2% O_2_ (White Martins, Itapira, SP, Brazil)) for WT and TRPV1KO mice).

### 2.3. Mechanical Hypernociceptive Responses

The ability of CHIKV and its RNA to cause nociception was initially assessed. For this, anaesthetized mice (n = 3 males and 2 females/group) received either an intra-articular injection containing inactivated CHIKV (10^6^ PFUs/joint) or its RNA (50 ng/joint) into the mouse ipsilateral knee joint, and the contralateral joint received an equal volume of sterile saline (10 µL/joint; Samtec Biotecnologia, Ribeirão Preto, SP, Brazil). Secondary hypernociceptive responses to mechanical stimuli were evaluated in the ipsilateral and contralateral hindpaws by the up-and-down method by using von Frey filaments [22]. Heat-induced responses were determined by the hot plate test [23]. For this, mice were scruffed loose, and each hindpaw was individually placed on a metal surface set at 45 °C. Latency to a nociceptive response (licking or shaking of hind paws, jumping) was registered for each hindpaw, with a 2 min interval between measures; a cut-off of 20 sec was used to prevent tissue damage. For comparison, a group of animals (n = 3 males and 2 females) received only saline in the ipsilateral joint and no injections in the contralateral side. Both mechanical and heat nociceptive responses were analyzed at baseline and 3 and 7 days post-i.art. injections.

In a separate set of experiments, mice were i.art. injected with CHIKV or RNA and 7 days later (n = 12 males and 8 females/group) received either vehicle (5 mL/kg, sterile saline plus 6% dimethyl sulfoxide (DMSO); n = 3 males and 2 females) or the selective TRPV1 antagonist SB366791 (0.5 mg/kg, s.c.; n = 3 males and 2 females; Sigma-Aldrich) [19]. After 1 and 4 h, the nociceptive responses were measured and compared with baseline and pre-treatment readings. TRPV1 WT (n = 18 males and 18 females) and KO (n = 18 males and 18 females) mice received i.art. CHIKV or RNA in the ipsilateral knee joint and sterile saline in the contralateral side; their nociceptive responses were evaluated at baseline and on the 7th day post-injection, and compared with those of mice i.art. injected with saline in the ipsilateral joint (n = 3 males and 3 females).

### 2.4. Knee Joint Histology

Knee joint samples (contra- and ipsilateral) of TRPV1 WT and KO mice were collected at 7 days post-CHIKV (10^6^ PFUs/joint), RNA (50 ng/joint) or saline (10 µL) i.art. injections (n = 3 males and 3 females/group) in paraformaldehyde 10%, decalcified in EDTA solution (5%; 4 °C, for 2-months, solution replaced at every 14 days; Sigma-Aldrich) and embedded in paraffin. Then, 5 µm sagittal sections were cut on a microtome, deparaffinized in xylene, dehydrated in series of ethanol/water, and stained with hematoxylin and eosin. The sections were analyzed by using an Axio Scan.Z1Scanner (Zeiss, Jena, Germany; 200× magnification) and ZEN software (Blue Edition; Zeiss). Tissue morphology was evaluated by a blind investigator, and scores (0 = absence, 1 = mild, 2 = moderate and 3 = severe) were attributed as previously described [23] to synovial hypertrophy, cartilage damage, bone erosion and inflammation. The summation of all scores for each individual joint of each mouse was considered as the articular damage score.

### 2.5. TRPV1 Immunohistochemistry

The cartilage expression of TRPV1 was analyzed in contra- and ipsilateral knee joint sections (5 µm) of CHIKV- and saline-injected mice (n = 3 males and 2 females/group) at 7 days post-i.art. injections. Samples were deparaffinized and incubated with blocking buffer (10% goat serum, 1% bovine serum albumin, and 0.025% Triton X-100 in 1× Tris-buffered saline) for 2 h at room temperature. Then, samples were incubated with the anti-TRPV1 (rabbit polyclonal, ACC-030, 1:200; Alomone, Jerusalem, Israel) primary antibody, at 4 °C overnight. The slides were then incubated with the Mouse and Rabbit Specific HRP/DAB IHC Detection Kit according to the manufacturer’s instructions (ab236466; Abcam, Cambridge, UK). Images from each slide were taken (400× magnification; Zeiss, Jena, Germany), and the percentage (%) of positive area to each staining in the cartilage was analyzed by using Image-ProPlus software (version 4.5.0.29; Media Cybernetics, Silver Spring, MD, USA).

### 2.6. Statistical Analyses

Statistical analyses were performed on GraphPad Prism 8.0. Data obtained from the hypernociceptive tests are expressed as mean + standard error of the mean (S.E.M.) and were analyzed by repeated measures, 1-way or 2-way analysis of variance (ANOVA), followed by Bonferroni’s test. Histology and immunohistochemistry data are expressed in boxplots as median (minimum-maximum), and were analyzed by Kruskal–Wallis followed by Dunn’s test. *p* values < 0.05 were considered as significant differences between groups.

## 3. Results

### 3.1. TRPV1 Antagonism and Ablation Attenuate CHIKV- and Viral RNA-Induced Nociception

**Figure 1:**
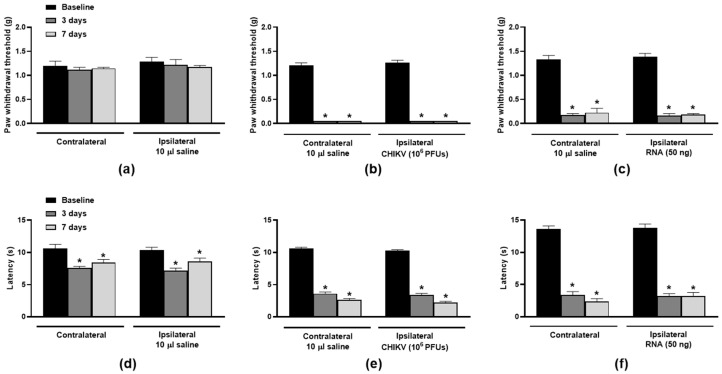
**CHIKV particle- and RNA-induced nociception in the mouse hindpaws.** Mechanical (**a**–**c**) and thermal (**d**–**f**) hindpaw sensitivities following saline (10 µL/joint), CHIKV particle (10^6^ PFUs/joint) or RNA (50 ng/joint) intra-articular injection in the ipsilateral joints. The contralateral joints of saline-injected mice were uninjected, whilst those of CHIKV- or RNA-injected mice received 10 µL saline. Paw withdrawal thresholds and latencies were measured at baseline and days 3 and 7 post-i.art. injections. * *p* < 0.05, differs from baseline measurements. n = 3 males and 2 females/group.

The nociceptive responses triggered by CHIKV or its RNA were initially assessed. The i.art. ipsilateral injection of CHIKV (10^6^ PFUs/joint) or its RNA (50 ng/joint) induced pronounced bilateral hindpaw hypersensitivities to mechanical (Figure 1a–c) and heat (Figure 1d–f) stimuli in comparison with ipsilateral saline-injected mice.

**Figure 2:**
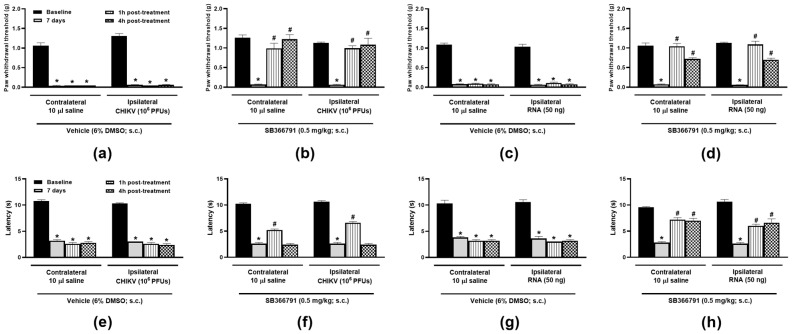
**TRPV1 antagonism reduces the hypersensitivity to mechanical and heat stimuli in CHIKV particle- or RNA-injected mice.** Mechanical (**a**–**d**) and thermal (**e**–**h**) hindpaw sensitivities following saline (10 µL/joint), CHIKV particle (10^6^ PFUs/joint) or RNA (50 ng/joint) intra-articular injection in the ipsilateral joints. The contralateral joints received 10 µL saline. Paw withdrawal thresholds and latencies were measured at baseline, 7 days post-i.art. injections, and at 1 and 4 h after SB366791 (0.5 mg/kg, s.c.) or vehicle (6% DMSO in saline) treatments. * *p* < 0.05, differs from baseline measurements. ^#^ *p* < 0.05, differs from measurements taken on day 7 post-i.art. injections. n = 3 males and 2 females/group.

**Figure 3:**
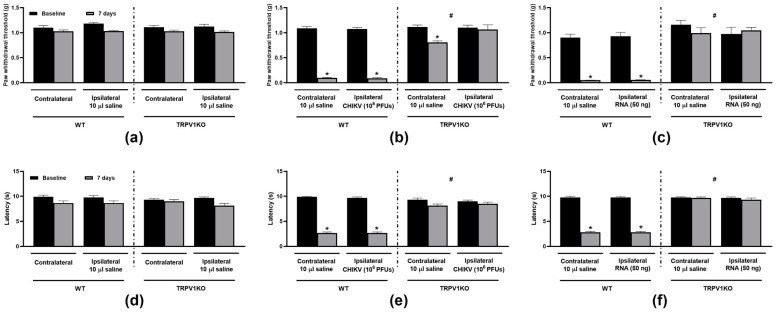
**TRPV1 ablation impairs the development of CHIKV particle- and RNA-induced mechanical and heat hypersensitivities.** Mechanical (**a**–**c**) and thermal (**d**–**f**) hindpaw sensitivities following saline (10 µL/joint), CHIKV particle (10^6^ PFUs/joint) or RNA (50 ng/joint) intra-articular injection in the ipsilateral joints of TRPV1 WT and KO mice. The contralateral joints of saline-injected mice were uninjected, whilst those of CHIKV- or RNA-injected mice received 10 µL saline. Paw withdrawal thresholds and latencies were measured at baseline and 7 days post-i.art. injections. * *p* < 0.05, differs from baseline measurements. ^#^ *p* < 0.05, differs from WT measurements. n = 3 males and 3 females/group.

Treatment with the TRPV1 antagonist (SB366791, 0.5 mg/kg, s.c.) reduced hypersensitivities to mechanical (Figure 2a–d) and heat (Figure 2e,f) stimuli in mice injected either with CHIKV particle or RNA in comparison with the vehicle (6% DMSO in saline, 5 mL/kg, s.c.). Whilst SB366791 produced a total reversal of the bilateral mechanical nociceptive responses to both CHIKV particle and RNA, only a partial increase in latency to heat was noted for those injected with either virus or RNA. Animals lacking TRPV1 did not develop bilateral mechanical or heat hypersensitivities when i.art.-injected with CHIKV particle or RNA (Figure 3b,c,e,f) at 7 days post-injection. Ipsilateral saline injection did not affect thresholds and latencies in TRPV1 WT or KO mice (Figure 3a,d).

### 3.2. TRPV1 Protein Expression in the Knee Joint Cartilage Is Induced by CHIKV

**Figure 4:**
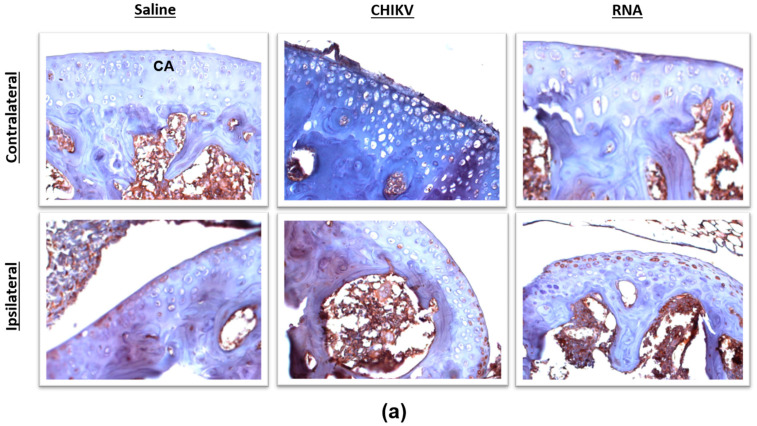

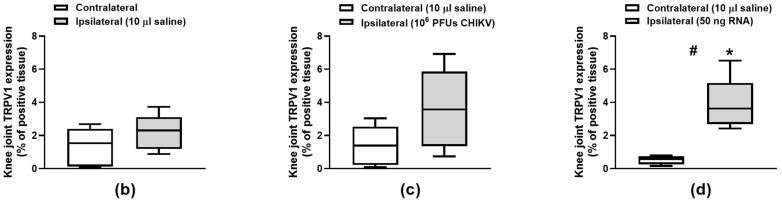
**CHIKV induces TRPV1 expression in the mouse knee joint cartilage.** (**a**) Representative panels (400 µm) of contralateral and ipsilateral mouse knee joints obtained at day 7 post-CHIKV (10^6^ PFUs/joint), -RNA (50 ng/joint) or -saline (10 µL/joint) injection in the ipsilateral joint. Percentage (%) of TRPV1 positive cartilage tissue from knee joints of (**b**) saline-, (**c**) CHIKV- or (**d**) RNA-injected mice. The contralateral joints of saline-injected mice were uninjected whilst those of CHIKV-injected mice received 10 µL saline. * *p* < 0.05, differs from the contralateral joint. ^#^ *p* < 0.05, differs from mice that received saline in the ipsilateral joints. n = 3 males and 2 females/group. Brown staining indicates TRPV1 expression. CA: cartilage tissue.

The ability of CHIKV to trigger changes in the pattern of TRPV1 protein expression in the knee joint was evaluated (Figure 4a–d). CHIKV and its RNA promoted an increase in TRPV1 expression in the ipsilateral knee joint cartilages; this effect was greater and significant in animals injected with RNA in comparison with their contralateral joints and those of saline-injected mice (Figure 4d).

### 3.3. CHIKV-Induced Histology Changes Are Not Affected by Loss of TRPV1 Function

**Figure 5:**
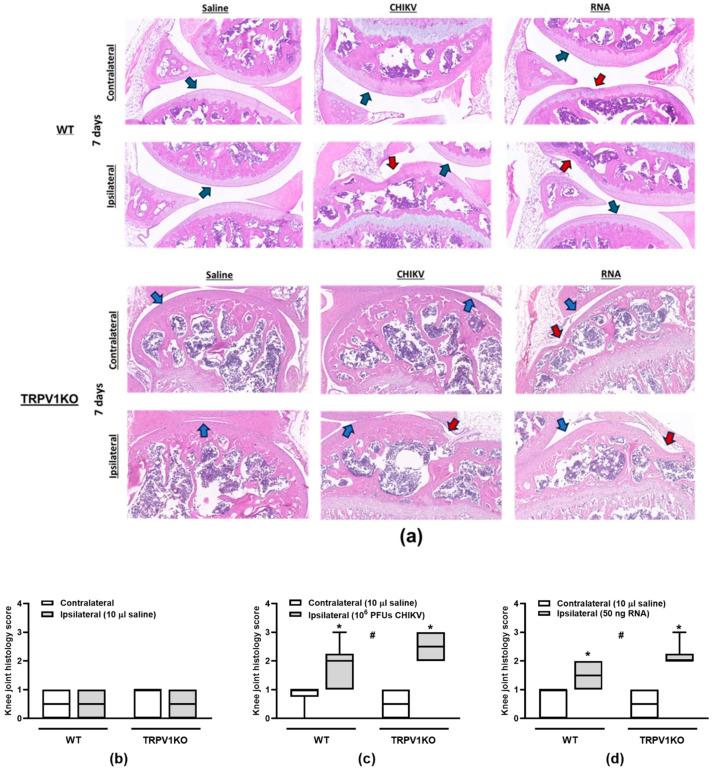
**TRPV1 ablation does not affect the knee joint histology scores in CHIKV particle- and RNA-injected mice.** (**a**) Representative panels (200 µm) of TRPV1KO and WT contralateral and ipsilateral mouse knee joints obtained at day 7 post-CHIKV (10^6^ PFUs/joint), -RNA (50 ng/joint) or -saline (10 µL/joint) injection in the ipsilateral joint. Total histology scores in the contralateral and ipsilateral knee joints from WT and TRPV1KO mice i.art. injected with (**b**) saline (10 µL/joint), (**c**) CHIKV particle (10^6^ PFUs/joint) or (**d**) RNA (50 ng/joint). The contralateral joints of saline-injected mice were uninjected, whilst those of CHIKV- or RNA-injected mice received 10 µL saline. * *p* < 0.05, differs from the contralateral joint. ^#^ *p* < 0.05, differs from mice that received saline in the ipsilateral joints. n = 3 males and 3 females/group. Red arrows represent loss of cartilage. Blue arrows represent normal cartilage tissue.

The impacts of TRPV1 ablation on the knee joint structures of mice i.art. injected with CHIKV particle, RNA or saline were assessed (Figure 5a–c). Regardless of genotype, both CHIKV and its RNA increased the total histology scores in the mouse ipsilateral joints (panels b and c) in comparison with their contralateral sides and saline-injected joints (panel a). The lack of functional TRPV1 did not alter the damaging effects of CHIKV particle or RNA in the mouse joints (Figure 5a–c).

## 4. Discussion

The deleterious actions of CHIVK virus in the joints and the resulting pain arising from them are well known with knee joints being greatly affected by this pathogen [24]. Despite the great advances in the last decade, including the recent advent of a CHIKV vaccine, a great parcel of the world’s population has been infected by the virus every year with a considerable number of individuals becoming affected by long-lasting joint and/or musculoskeletal pain [6,25].

The ability of CHIKV to cause hypersensitivity has been reproduced in rodent models with a range of common pathways to those of human disease, which have been described in the last few years; however, few have investigated the underlying mechanisms of pain. Herein, CHIKV and its RNA produced bilateral hypersensitivity, which is a response observed in infected patients [26] and in models in which sustained pain can be observed (TNFα-induced for example [23,27]). Recently, the contribution of central TRPV1 to CHIKV-induced pain was demonstrated in an in vivo model of hypersensitivity caused by the virus or its envelope protein E2 [14]. The authors found that CHIKV indirectly triggers the activation of TRPV1 in dorsal root ganglia neurones, contributing to hypersensitivity to mechanical and heat stimuli. These responses were present for 3 days, reaching their peaks in the first 7 h post-i.art. challenge with CHIKV or E2 protein. Herein, we present supporting evidence that TRPV1 is a key mediator of the nociceptive responses caused by CHIKV. The virus and its RNA induced sustained hypersensitivities to mechanical and heat stimuli (over 7 days). These responses were accompanied by an increased expression of TRPV1 in the cartilage and knee joint histologic changes. In a recent report, replicating CHIKV was shown to elicit the functional expression of TRPV1 on macrophages [15], constituting the first indication of the virus’s ability to induce this receptor on non-neuronal cells. Herein, we present the first evidence that both the inactivated virus and its RNA can enhance TRPV1 protein expression on cartilage cells, indicating CHIKV can cause a broader regulation of this nociceptive receptor on multiple cells, including peripheral tissues, even in the absence of an active infection (i.e., virus replication). It was also found that whilst the loss of TRPV1 function did not alter the impact of CHIKV in the knee joint tissues, TRPV1 is essential to both the generation and maintenance of sustained hypersensitivity responses triggered by the viral particle and RNA. The data are supported by previous findings in the acute response to the inactivated virus [14].

## 5. Conclusions

The data presented herein confirm the previously attributed role for TRPV1 in CHIVK-induced hypersensitivities to mechanical and heat stimuli and extend the knowledge on the importance of this receptor as a key transducer involved not only in the generation but also in the maintenance of nociception during the acute and sustained responses to CHIKV and its RNA. It also demonstrates for the first time the ability of the virus to enhance TRPV1 expression in the cartilage. This, in addition to previous reports of the capability of CHIKV to activate TRPV1 in macrophages and neurones, indicate that a complex regulation of different downstream pathways following this receptor activation may contribute to the painful disorder caused by the virus. Nonetheless, the importance of further studies to assess TRPV1 activation implications in CHIKV-induced human articular disease must be highlighted, since the existing differences between mouse and human immune systems [28,29,30] might result in the initiation of different cellular pathways. On the other hand, there is undeniable evidence on the importance of TRPV1 as a pain transducer in humans [11,12], although the use of receptor antagonists to treat painful disorders has proven to still be a clinical challenge [31].

## Data Availability

The original contributions presented in the study are included in the article, further inquiries can be directed to the corresponding author.
